# Development of a Loop-Mediated Isothermal Amplification Method for Rapid and Visual Detection of Monkeypox Virus

**DOI:** 10.1128/spectrum.02714-22

**Published:** 2022-09-26

**Authors:** Junxia Feng, Guanhua Xue, Xiaohu Cui, Bing Du, Yanling Feng, Jinghua Cui, Hanqing Zhao, Lin Gan, Zheng Fan, Tongtong Fu, Ziying Xu, Shuheng Du, Yao Zhou, Rui Zhang, Hanyu Fu, Ziyan Tian, Qun Zhang, Chao Yan, Jing Yuan

**Affiliations:** a Department of Bacteriology, Capital Institute of Pediatrics, Beijing, China; University of Arizona/Banner Health

**Keywords:** monkeypox virus, loop-mediated isothermal amplification, rapid detection, visual detection, orthopoxvirus

## Abstract

Monkeypox virus (MPXV) is a human pathogenic virus that belongs to the genus *Orthopoxvirus*. In 2022, MPXV caused an unprecedented number of infections in many countries. As it is difficult to distinguish MPXV from other pathogens by its symptoms in the early stage of infection, a rapid and reliable assay for MPXV detection is needed. In this study, we developed a loop-mediated isothermal amplification (LAMP) assay for the specific detection of MPXV and evaluated its application in simulated clinical samples. The A27L-1 and F3L-1 primer sets were identified as the optimal primers, and 63°C was the most appropriate reaction temperature for sequence amplification. The detection limits of the LAMP assay using primer sets A27L-1 and F3L-1 were both 20 copies/reaction mixture, which were >100-fold higher in terms of sensitivity, compared with conventional PCR. The LAMP assay findings were negative for all 21 non-MPXV pathogens, confirming the high specificity of our assay. All three types of simulated clinical samples were clearly identified by our LAMP assay, and the detection limits were consistent with the sensitivity results, indicating efficient clinical sample identification. Our rapid and reliable MPXV LAMP assay could be useful for MPXV detection and on-site diagnosis, especially in primary hospitals and rural areas.

**IMPORTANCE** MPXV outbreaks rapidly grew in the first half of 2022, and this virus has been recognized as an increasing public health threat, particularly in the context of the COVID-19 pandemic. Thus, developing reliable and fast detection methods for MPXV is necessary.

## INTRODUCTION

Monkeypox virus (MPXV) is a linear double-stranded DNA virus which belongs to the genus *Orthopoxvirus* in the family *Poxviridae*; it is a near-origin virus with the now-eradicated smallpox virus. MPXV isolates are divided into two clades, Congo Basin and West African; members of the Congo Basin clade are more virulent. MPXV infection occurs mostly in Central and West Africa; it was initially discovered in laboratory monkeys in 1958 (1). MPXV can be transmitted from animals to humans through direct contact with (or a bite from) an infected animal; it can also be directly transmitted among humans. The first human case of MPXV infection was detected in 1970 in the Democratic Republic of the Congo (2). The number of human cases of MPXV infection has been increasing since the 1970s; MPXV first appeared in the Western Hemisphere in 2003, when 82 cases of MPXV infection were reported in the United States (3). The increase in MPXV outbreaks worldwide may be related to the decline in human immunity to smallpox virus and other orthopoxviruses, which occurred after the World Health Organization announced the elimination of smallpox in 1980 and began to discontinue smallpox vaccination programs. Disturbingly, MPXV outbreaks have shown rapid growth in the first half of 2022, such that more than 1,400 cases and 66 deaths have been reported in African nations; more than 1,000 infections have been confirmed in nearly 30 countries where monkeypox virus outbreaks do not normally occur (4, 5). As the global coronavirus disease 2019 (COVID-19) pandemic continues, outbreaks of MPXV contribute to increased public health threats; thus, rapid detection of MPXV is needed to control its spread and enable timely treatment.

Loop-mediated isothermal amplification (LAMP) is a simple, rapid, and technology-independent detection method which uses *Bst* DNA polymerase to realize autocycling strand displacement of nucleic acids. The LAMP method was first developed by Notomi et al (6). Thus far, LAMP-based diagnosis has been widely developed for the detection of various pathogens, including viruses (7–13), bacteria (14, 15), parasites (16, 17), and fungi (18).

In this study, we established a LAMP detection method for rapid identification of MPXV. We designed five primer sets targeting the *A27L* or *F3L* genes and then selected the optimal primer sets and reaction temperature. Additionally, we determined the sensitivity and specificity of this method, and then we assessed its usefulness by evaluation of simulated clinical samples.

## RESULTS

### Optimal reaction conditions for MPXV pseudovirus detection by LAMP.

**FIG 1:**
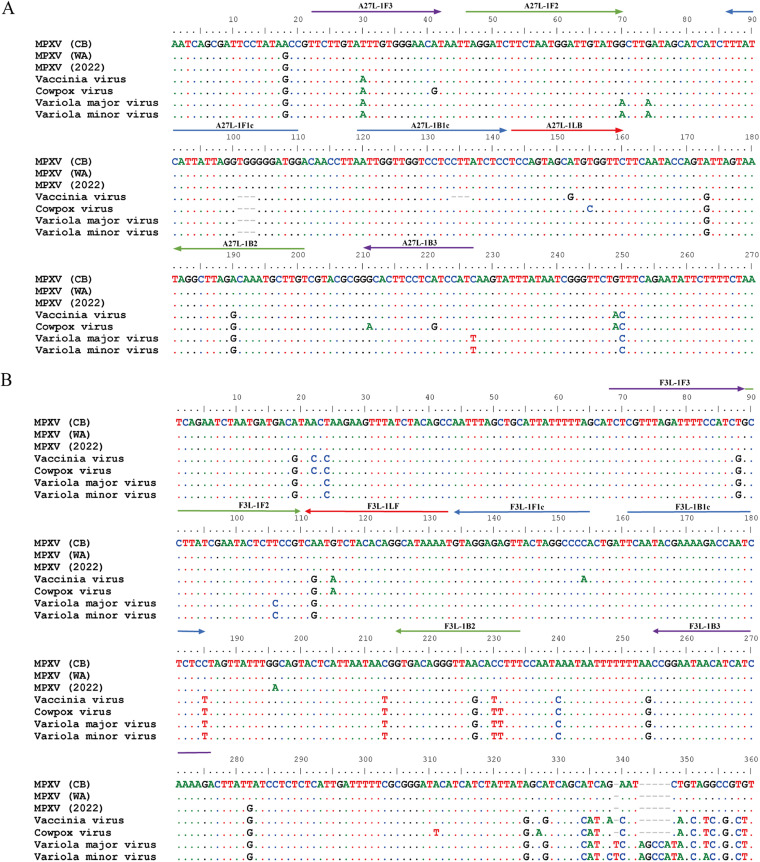
Primer sequence alignment with orthopoxvirus DNA. The primer sets A27L-1 and F3L-1 were aligned with the targeted sequence of DNA within several orthopoxvirus species. Virus strains: monkeypox (MPXV) West African strain MPXV-Singapore 2019 (MT250197), MPXV Congo Basin strain MPXV-Zaire-96-I-16 (GenBank AF380138); MPXV isolate Monkeypox/PT0001/2022 (GenBank ON585029); vaccinia virus Acambis/2019 (GenBank MT227314); cowpox virus CPXV/Cepad 332 (GenBank MK035759); variola major virus Bangladesh-1975 (GenBank L22579); variola minor virus (GenBank Y16780). (A) Sequence alignment of primer set A27L-1 with orthopoxvirus DNA; (B) Sequence alignment of primer set F3L-1 with orthopoxvirus DNA. CB, Congo Basin; WA, West African; F3, outer forward primer; B3, outer backward primer; FIP, forward inner primer F1c-F2; BIP, backward inner primer B1c-B2; LF, loop forward primer; LB, loop backward primer.

**FIG 2:**
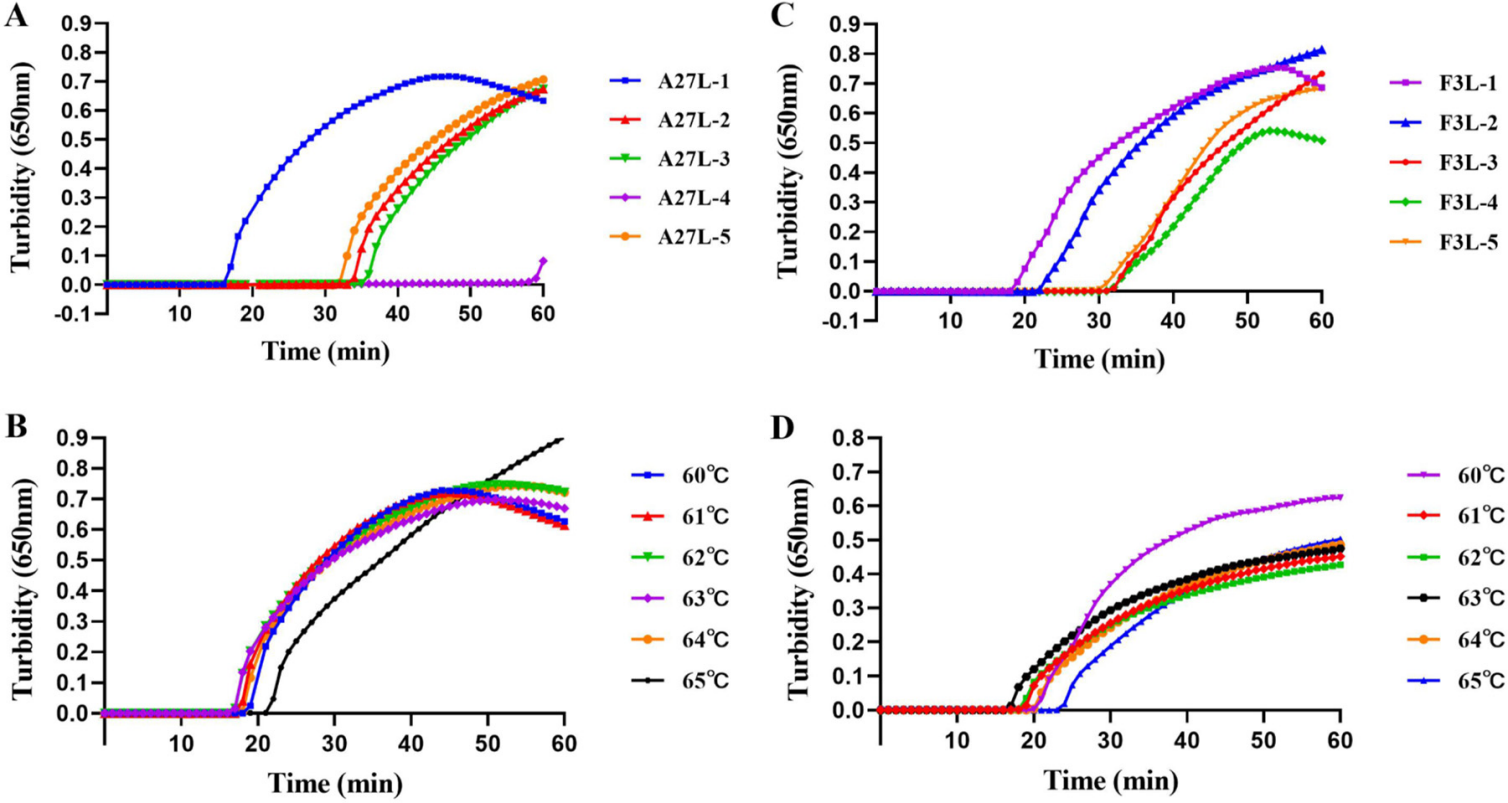
Analysis of optimal primers and reaction temperature for LAMP assay. (A) The best primer set for amplification of the *A27L* gene in the LAMP assay. (B) The optimal reaction temperature for the LAMP assay with the A27L-1 primer set. (C) The best primer set for amplifying *F3L* in the LAMP assay. (D) The optimal reaction temperature for the LAMP assay with the primer set F3L-1.

Conserved regions of the *A27L* and *F3L* genes of MPXV were selected as target sequences for primer design because of their 100% identity within MPXV but low identity relative to other orthopoxviruses (Fig. 1). DNA samples isolated from MPXV pseudovirus containing the target sequences were used as templates for the exploration of optimal LAMP assay conditions. Five sets of primers were designed (see Table S1 in the supplemental material) and tested for their abilities to detect the *A27L* gene (A27L-1, A27L-2, A27L-3, A27L-4, and A27L-5) and the *F3L* gene (F3L-1, F3L-2, F3L-3, F3L-4, and F3L-5) of MPXV pseudoviruses. As shown in Fig. 2, the primer sets A27L-1 and F3L-1 most rapidly initiated the LAMP amplification reaction under the same reaction temperature (63°C); these primer sets were used for further LAMP assays to detect MPXV pseudoviruses containing *A27L* and *F3L* genes.

To identify the optimal reaction temperature, the LAMP assay was carried out at 1°C temperature intervals, over the range from 60°C to 65°C. As shown in Fig. 2, 63°C was the most appropriate temperature for LAMP amplification of MPXV pseudoviruses containing *A27L* and *F3L* genes; this temperature was therefore used in all subsequent assays.

### LAMP assay sensitivity.

**FIG 3:**
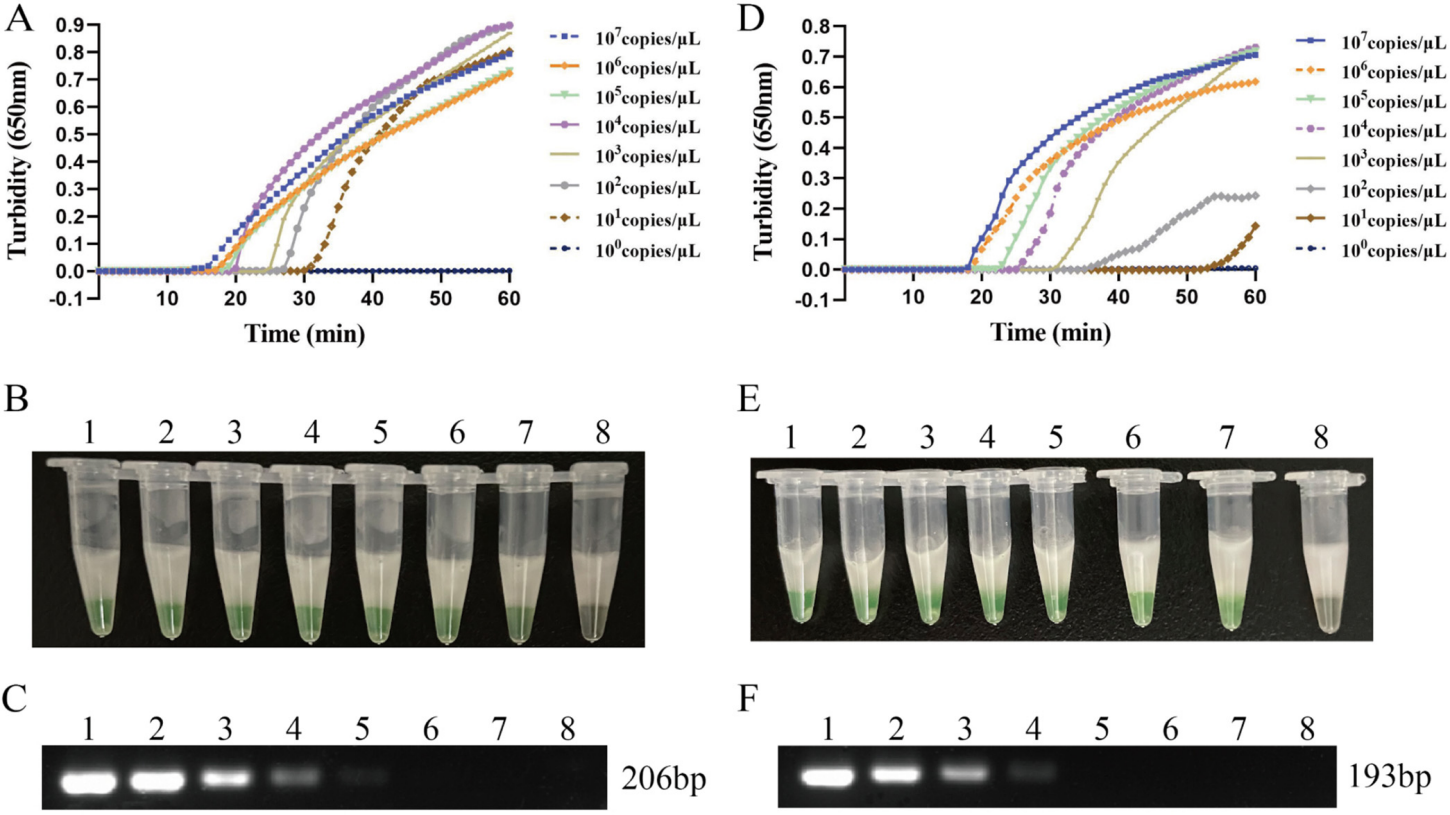
LAMP assay sensitivity for MPXV. (A and B) The sensitivity of LAMP reaction with primer set A27L-1, detected by turbidity and visual observation. (D and E) The sensitivity of LAMP reaction with primer set F3L-1, detected by turbidity and visual observation. (C and F) The sensitivity of the conventional PCR assay using primers that target the *A27L* and *F3L* genes of MPXV. The turbidity was measured using a Loopamp real-time turbidimeter, and the result also was detected by visual observation according to the color change from orange to green. Five-microliter volumes of PCR products of conventional PCR were separated by 1% agarose gel electrophoresis, and samples positive for the MPXV *A27L* gene showed a DNA fragment at 206 bp, while those positive for the *F3L* gene showed a DNA fragment at 193 bp. Lanes: 1, 10^7^ copies/μL; 2, 10^6^ copies/μL; 3, 10^5^ copies/μL; 4, 10^4^ copies/μL; 5, 10^3^ copies/μL; 6, 10^2^ copies/μL; 7, 10^1^ copies/μL; 8, 10^0^ copies/μL.

DNA of MPXV pseudovirus containing the target sequence of *A27L* and *F3L* was extracted using a QIAamp DNA minikit (Qiagen, Hilden, Germany) and was diluted 10-fold to concentrations ranging from 10^7^ copies/μL to 1 copy/μL; this DNA was used as a template for the assessment of LAMP assay sensitivity. As shown in Fig. 3, the time required for positive detection with primer set A27L-1 ranged from 16 min at 1 × 10^7^ copies/μL to 31 min at 1 × 10^1^ copies/μL; the time required for positive detection with primer set F3L-1 ranged from 18 min at 1 × 10^7^ copies/μL to 55 min at 1 × 10^1^ copies/μL. The findings of the turbidimeter assay and the naked-eye color assay were nearly identical. The detection limits of the primer set A27L-1 and F3L-1 were both 20 copies/reaction mixture. The sensitivities of conventional PCR for the *A27L* and *F3L* genes were 2 × 10^3^ and 2 × 10^4^ copies/reaction mixture.

### LAMP assay specificity.

**FIG 4:**
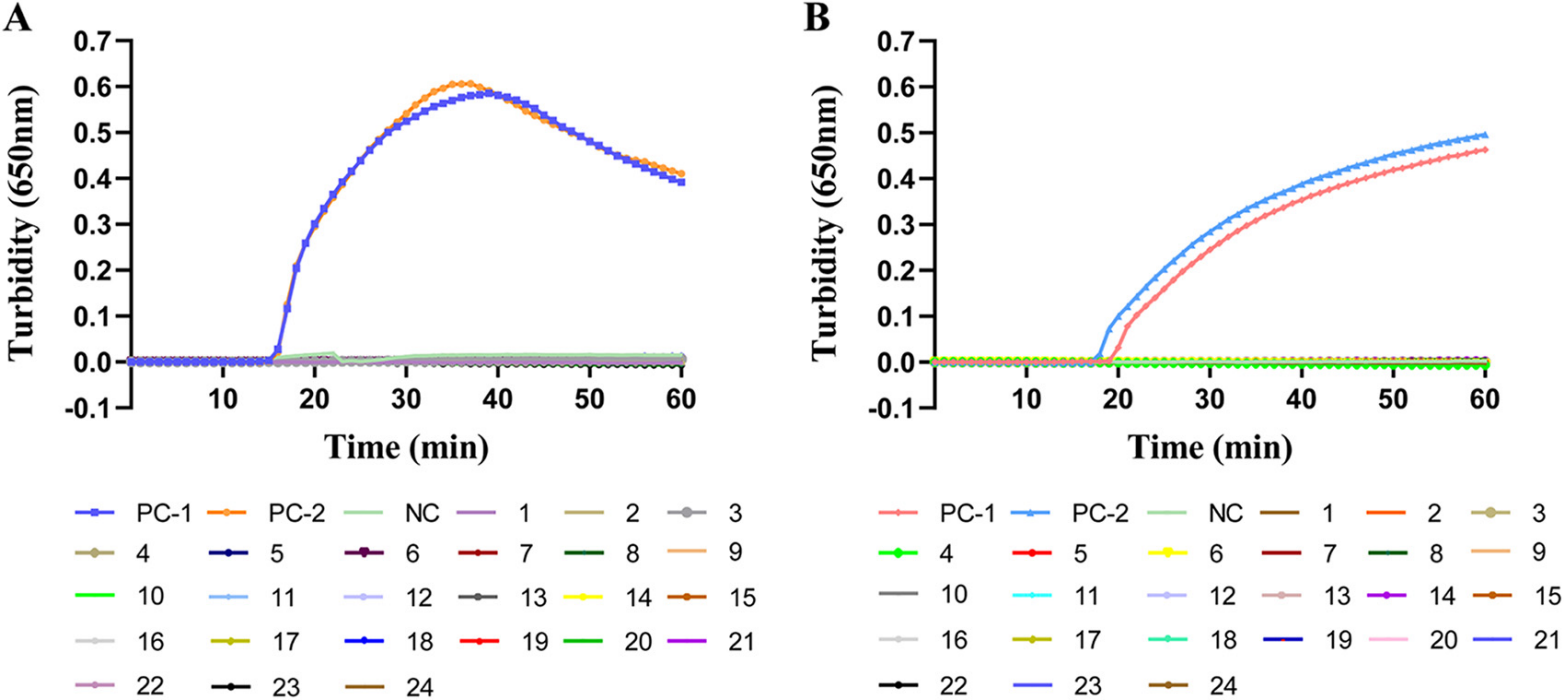
LAMP assay specificity for MPXV. (A) The specificity of the LAMP reaction with primer set A27L-1, detected by turbidity. Color key: PC-1, positive control (MPXV pUC-A27L recombinant plasmid); PC-2, positive control (pseudovirus MPXV-A27L); NC, negative control (sterile water); 1, variola virus plasmid containing A27L fragment; 2, cowpox virus plasmid containing A27L fragment; 3, vaccinia virus plasmid containing A27L fragment; 4, human coronavirus; 5, influenza B virus; 6, respiratory syncytial virus A; 7, respiratory syncytial virus B; 8, parainfluenza virus; 9, human metapneumovirus; 10, human enterovirus 71; 11, coxsackievirus A; 12, coxsackievirus B; 13, herpes simplex virus 1; 14, herpes simplex virus type 2; 15, varicella-zoster virus; 16, adenovirus; 17, human bocavirus; 18, rhinovirus; 19, Klebsiella pneumoniae; 20, Mycoplasma pneumoniae; 21, Streptococcus pneumoniae; 22, Haemophilus influenzae; 23, Stenotrophomonas maltophilia; 24, Staphylococcus aureus. (B) The specificity of the LAMP reaction with primer set F3L-1, detected by turbidity. The turbidity was measured using a Loopamp real-time turbidimeter. Color key: PC-1, positive control (MPXV pUC-F3L recombinant plasmid); PC-2, positive control (pseudovirus MPXV-F3L); NC, negative control (sterile water); 1, variola virus plasmid containing F3L fragment; 2, cowpox virus plasmid containing F3L fragment; 3, vaccinia virus plasmid containing F3L fragment (remaining color assignments 4 to 24 are those listed for panel A).

To evaluate the specificity of our LAMP assay, the recombinant plasmids pUC57-A27L and pUC57-F3L, as well as pseudovirus at a concentration of 1 × 10^7^ copies/μL, were used as templates; sterile water was used as the negative control. The same concentration for 21 other human pathogens was also examined. The LAMP assay with primer sets A27L-1 and F3L-1 revealed positive results for only the positive control sample, according to turbidity monitoring and visual observation; the results were negative for all other pathogens, as well as for the blank control (Fig. 4). Thus, the LAMP assay was specific for MPXV pseudoviruses and plasmids containing *A27L* or *F3L* genes.

### Detection of simulated clinical samples.

Simulated clinical samples were prepared to explore the feasibility of our LAMP assay for clinical detection. Clinical samples that were not mixed with pseudovirus were used as negative controls. The simulated clinical samples were analyzed using the LAMP and PCR assays. As shown in Table 1, MPXV pseudoviruses containing *A27L* and *F3L* genes were detected in the simulated peripheral blood, throat swab, and skin swab samples by both LAMP and conventional PCR. The minimal detectable concentrations in simulated peripheral blood and skin swab samples were 2 × 10^1^ copies/reaction mixture by LAMP utilizing either primer set (A27L-1 or F3L-1). The minimal detectable concentration in throat swabs was 2 × 10^1^ copies/reaction mixture using primer set A27L-1 and 2 × 10^2^ copies/reaction mixture using primer set F3L-1. MPXV pseudoviruses containing *A27L* and *F3L* genes in simulated clinical samples were also detectable by conventional PCR, with higher minimal detectable concentrations for both *A27L* and *F3L* (Table 1; see also Fig. S2 in the supplemental material).

**TABLE 1:** Results of the simulated clinical sample test using LAMP compared to PCR

Simulated clinical sample	LAMP assay MDC^*a*^		Conventional PCR MDC^*a*^	
	A27L	F3L	A27L	F3L
Peripheral blood	2 × 10^1^	2 × 10^1^	2 × 10^3^	2 × 10^4^
Skin swab	2 × 10^1^	2 × 10^1^	2 × 10^3^	2 × 10^4^
Throat swab	2 × 10^1^	2 × 10^2^	2 × 10^3^	2 × 10^5^

a Minimal detectable concentration (MDC), in copies per reaction mixture.

## DISCUSSION

MPXV is one of four orthopoxviruses that are pathogenic to humans; the others are variola virus, vaccinia virus, and cowpox virus. Concerns have recently been raised regarding the emergence of MPXV (4, 19), as well as its clinical similarity to smallpox, a devastating illness that was eliminated 40 years ago by vaccination. MPXV has been recognized as an increasing public health threat, particularly in the context of the COVID-19 pandemic. Thus, there is a need to develop a rapid detection method to control the spread of MPXV. Since 2006, several real-time PCR methods have been developed to detect MPXV (20–22). This well-developed PCR method is useful for laboratory analysis because of its high sensitivity and specificity; however, it is not ideal for primary hospitals and rural regions, particularly during disease outbreaks. Thus, a rapid and simple detection method is important for controlling infectious disease spread.

Compared with conventional detection techniques, LAMP assays have several advantages, including high specificity, high sensitivity, simplicity, and low cost. Notably, a LAMP assay can be performed at a constant temperature (6), and the results can be visually observed by color changes in the reaction mixture; expensive experimental conditions and complicated procedures are unnecessary. Most of the above-mentioned advantages cannot be matched by other molecular biological methods. In this study, we developed and optimized a LAMP assay for rapid and efficient detection of MPXV and then compared it with conventional PCR.

Since MPXV was first isolated in 1970, it has evolved into two distinct clades: Congo Basin and West African (3). Members of the Congo Basin clade usually cause more severe disease (case fatality rate of 10.6%) and are more easily transmitted. The MPXV genome changes with viral spread; recent epidemic MPXV isolates are within the West African clade but display unique sequences (23). Furthermore, MPXV has substantial similarity to the other four orthopoxviruses that are pathogenic to humans; thus, appropriate primers are important for LAMP assay specificity.

In this study, we selected the conserved regions of two target genes of MPXV (*F3L* and *A27L*) for LAMP primer design. The *F3L* gene is commonly used for real-time detection (22), while the *A27L* gene was selected to improve amplification specificity in this study. We compared 168 complete virus genomes and designed the specific primers A27L-1 and F3L-1 in the region conserved among all MPXVs, which was clearly distinct from genomes of other orthopoxviruses. When these primers were used for the detection of 21 other pathogens, we found no cross-reactivity, indicating ideal specificity for the target virus.

To examine the sensitivity of established LAMP methods, we used MPXV pseudoviruses containing the target sequence. Pseudoviruses are more likely than plasmids to clearly indicate the effectiveness of the assay. The primers A27L-1 and F3L-1 both had a detection limit of 20 copies/μL in the LAMP assay of MPXV pseudoviruses containing *A27L* and *F3L* genes; these were 100- to 1,000-fold more sensitive than the conventional PCR assay. The high level of sensitivity of our LAMP assay was comparable to the sensitivity achieved with the real-time PCR assay for MPXV (22). Iizuka et al. constructed a LAMP assay to identify Congo Basin and West African MPXV; its sensitivity was approximately 10^2^ to 10^3^ copies/reaction mixture, and our LAMP primers exhibited higher sensitivity (24). To verify the MPXV LAMP assay for clinical use, we prepared simulated clinical samples. MPXV is presumed to spread from close contact with bodily fluids (4), so we mixed peripheral blood, skin swab, and throat swab samples with various concentrations of MPXV pseudoviruses containing F3L and A27L to generate simulated clinical samples; each simulated sample was examined using the LAMP assay and conventional PCR. All three types of simulated samples were detectable by both assays. Our estimation of theoretical sample copies indicated that the detection limit of each type of simulated sample was consistent with the sensitivity results.

In summary, our LAMP assay can effectively detect MPXV pseudoviruses containing *F3L* and *A27L* genes, at a minimum concentration of 20 copies/reaction mixture, using primer set A27L-1 and F3L-1 with an optimal temperature of 63°C. This MPXV LAMP assay provides a useful tool to facilitate the surveillance and clinical diagnosis of MPXV.

## MATERIALS AND METHODS

### Viruses and DNA samples.

MPXV pseudoviruses MPXV-A27L and MPXV-F3L constructed from replication-deficient human type 5 adenovirus (Ad5) were purchased from Sangon Biotech Co., Ltd. (Shanghai, China). Pseudovirus was diluted 10-fold from 1 × 10^8^ copies/μL to 1 copy/μL for use in sensitivity tests. Human coronavirus, influenza B virus, respiratory syncytial virus A/B, parainfluenza virus, human metapneumovirus, human enterovirus 71, coxsackievirus A, coxsackievirus B, herpes simplex virus 1, herpes simplex virus type 2, varicella-zoster virus, adenovirus, human bocavirus, rhinovirus, Klebsiella pneumoniae, Mycoplasma pneumoniae, Streptococcus pneumoniae, Haemophilus influenzae, Stenotrophomonas maltophilia, and Staphylococcus aureus isolates were from our laboratory stocks. DNA was extracted using QIAamp DNA minikit (Qiagen, Hilden, Germany), in accordance with the manufacturer’s instructions.

### Plasmid construction.

A 500-bp sequence of the *A27L* gene (positions 136708 to 137207) from MPXV (GenBank accession number AF380138) was synthesized, and the EcoRI and HindIII restriction sites were incorporated into the *A27L* flank gene. The pUC57 vector and the synthesized *A27L* gene fragment were then digested with the EcoRI and HindIII enzymes, and the *A27L* gene was cloned into the vector through a ligation reaction to obtain the recombinant plasmid pUC57-A27L. The plasmid pUC57-F3L, containing the full length of the 462-bp *F3L* gene (positions 48048 to 48509), was obtained using the same method. The homologous regions of the *A27L* and *F3L* genes from vaccinia virus (GenBank MT227314), variola virus (GenBank LR800245), and cowpox virus (GenBank MK035759) were also synthesized and cloned into the pUC57 vector for use in specificity tests. The gene sequences were synthesized by Sangon Biotech Co., Ltd. The copy number was calculated using the following formula: DNA copy number, in copies per microliter = [(6.02 × 10^23^) × (plasmid concentration, in nanograms per microliter) × 10^−9^]/[(fragment length, in nucleotides) × 660].

### Primer design.

A total of 168 genome sequences of MPXV were acquired from the NCBI GenBank database and aligned to ensure that the selected sequences were specific to MPXV.

BLAST software (National Center for Biotechnology Information) was used for homology analysis. The *A27L* and *F3L* genes were selected as the target sequences; primers were designed using the software PrimerExplorer v5, available at http://primerexplorer.jp/e/. Five or six primers were generated for each gene, including two outer primers (F3 and B3), a forward inner primer (FIP), a backward inner primer (BIP), a loop forward primer (LF) and/or a loop backward primer (LB). All primers were first assessed by using OligoEvaluator software for primer dimer and hairpin structure and then submitted for a BLAST search to ensure alignment was exclusive to the desired target genes (https://blast.ncbi.nlm.nih.gov/Blast.cgi). The designed primers were commercially synthesized (Sangon Biotech Co., Ltd.) and purified by high-performance liquid chromatography.

### LAMP assay system.

The LAMP assay was performed using the Loopamp DNA amplification kit (Eiken Chemical Co., Ltd., Tochigi, Japan). Each 25-μL reaction mixture was prepared with 2 μL of DNA template, 12.5 μL of 2× reaction mix, 1 μL of *Bst* DNA polymerase, 1 μL of fluorescence detection reagent, 0.4 μL of FIP (100 μM), 0.4 μL of BIP (100 μM), 0.2 μL of LB/LF (100 μM), 0.05 μL of F3 (100 μM), 0.05 μL of B3 (100 μM), and sterile water up to 25 μL. The mixtures were added to reaction tubes and covered with a refined paraffin wax (25) to prevent cross-contamination by aerosols. Amplification was then performed on a Loopamp real-time turbidimeter (LA-320C; Eiken Chemical Co., Ltd., Tochigi, Japan) at 63°C for 60 min. Turbidity readings at an optical density of 650 nm were collected at 6-s intervals to monitor real-time changes in turbidity. A turbidity value of >0.1 was regarded as a positive result. In the visual assay, 1 μL of fluorescent calcein was added to the mixture before each reaction; the color of the reaction mixture changed from orange to green in positive samples, and this change was visible to the naked eye.

### PCR assay.

A conventional PCR assay was used as the reference standard for the LAMP assay. Five primer pairs were selected and tested for their abilities to detect the *A27L* gene and the *F3L* gene of MPXV pseudovirus. Optimal primers for *A27L* (A27L-1F3 and A27L-1B3) and *F3L* (F3L-1F3 and F3L-1B3) and the reaction temperature (60°C) were identified for conventional PCR assay (see Fig. S1 in the supplemental material). The PCR mixture contained the following components: 12.5 μL of PCR mix reagents (Tiangen Biotech Co., Ltd., Beijing, China), 0.3 μL forward primer (10 μM), 0.3 μL reverse primer (10 μM), 9.9 μL sterile water, and 2 μL DNA template (identical to the volume in the LAMP assay). Amplification was performed on a thermal cycler (Veriti 96-well thermal cycler, Applied Biosystems) with the following cycling profile: denaturation at 95°C for 10 min; 40 cycles of denaturation at 94°C for 30 s, annealing at 60°C for 45 s, and extension at 72°C for 45 s; and final extension at 72°C for 3 min. Amplified PCR products (5-μL volumes) were separated by 1% agarose gel electrophoresis (120 V, 25 min) and stained with GelGreen. Images were taken using a gel imaging system (GelDoc TM XR+ imager, Bio-Rad Laboratories, Co., Ltd.). Samples positive for the MPXV *A27L* or *F3L* gene showed a DNA ladder of ~200 bp.

### Preparation of simulated clinical samples.

Eight human skin swabs, 8 throat swabs, and 8 peripheral blood samples were collected from 24 individuals without orthopoxvirus infection, and each skin swab or throat swab was fully immersed in 2 mL of virus transport medium. The MPXV pseudoviruses MPXV-A27L and MPXV-F3L were mixed in equal amounts to a concentration of 4 × 10^8^ copies/μL. The pseudovirus mixture was then diluted 10-fold into eight concentrations (from 4 × 10^7^ to 4 × 10° copies/μL), and 100 μL of each dilution was mixed with 100 μL of skin swab solution, throat swab solution, or peripheral blood and then subjected to DNA extraction following the QIAamp DNA minikit protocol (Qiagen, Hilden, Germany). The resulting DNA extracts were used as simulated clinical sample templates for LAMP and PCR assays.

### Ethics statement.

This work was approved by the research board of the Ethics Committee of the Capital Institute of Pediatrics and was performed in compliance with the Helsinki Declaration (Ethical Principles for Medical Research Involving Human Subjects).

## References

[1] von Magnus P, Andersen EK, Petersen KB, Birch-Andersen A. 2009. A pox-like disease in cynomolgus monkeys. Acta Pathol Microbiol Scand 46:156–176. doi:10.1111/j.1699-0463.1959.tb00328.x.

[2] Ladnyj ID, Ziegler P, Kima E. 1972. A human infection caused by monkeypox virus in Basankusu Territory, Democratic Republic of the Congo. Bull World Health Organ 46:593–597. 4340218 PMC2480792

[3] Likos AM, Sammons SA, Olson VA, Frace AM, Li Y, Olsen-Rasmussen M, Davidson W, Galloway R, Khristova ML, Reynolds MG, Zhao H, Carroll DS, Curns A, Formenty P, Esposito JJ, Regnery RL, Damon IK. 2005. A tale of two clades: monkeypox viruses. J Gen Virol 86:2661–2672. doi:10.1099/vir.0.81215-0. 16186219

[4] Kozlov M. 2022. Monkeypox goes global: why scientists are on alert. Nature 606:15–16. doi:10.1038/d41586-022-01421-8. 35595996

[5] Kozlov M. 2022. Monkeypox vaccination begins: can the global outbreaks be contained? Nature 606:444–445. doi:10.1038/d41586-022-01587-1. 35676362

[6] Notomi T, Okayama H, Masubuchi H, Yonekawa T, Watanabe K, Amino N, Hase T. 2000. Loop-mediated isothermal amplification of DNA. Nucleic Acids Res 28:E63. doi:10.1093/nar/28.12.e63. 10871386 PMC102748

[7] Castro T, Sabalza M, Barber C, Abrams W, Da Costa AC, De Padua Milagres FA, Braz-Silva PH, Malamud D, Gallottini M. 2018. Rapid diagnosis of Zika virus through saliva and urine by loop-mediated isothermal amplification (LAMP). J Oral Microbiol 10:1510712. 30202506 10.1080/20002297.2018.1510712 PMC6127837

[8] Huang P, Wang H, Cao Z, Jin H, Chi H, Zhao J, Yu B, Yan F, Hu X, Wu F, Jiao C, Hou P, Xu S, Zhao Y, Feng N, Wang J, Sun W, Wang T, Gao Y, Yang S, Xia X. 2018. A rapid and specific assay for the detection of MERS-CoV. Front Microbiol 9:1101. doi:10.3389/fmicb.2018.01101. 29896174 PMC5987675

[9] Huang WE, Lim B, Hsu CC, Xiong D, Wu W, Yu Y, Jia H, Wang Y, Zeng Y, Ji M, Chang H, Zhang X, Wang H, Cui Z. 2020. RT-LAMP for rapid diagnosis of coronavirus SARS-CoV-2. Microb Biotechnol 13:950–961. doi:10.1111/1751-7915.13586. 32333644 PMC7264870

[10] Lopez-Jimena B, Bekaert M, Bakheit M, Frischmann S, Patel P, Simon-Loriere E, Lambrechts L, Duong V, Dussart P, Harold G, Fall C, Faye O, Sall AA, Weidmann M. 2018. Development and validation of four one-step real-time RT-LAMP assays for specific detection of each dengue virus serotype. PLoS Negl Trop Dis 12:e0006381. doi:10.1371/journal.pntd.0006381. 29813062 PMC5973574

[11] Parida M, Posadas G, Inoue S, Hasebe F, Morita K. 2004. Real-time reverse transcription loop-mediated isothermal amplification for rapid detection of West Nile virus. J Clin Microbiol 42:257–263. doi:10.1128/JCM.42.1.257-263.2004. 14715762 PMC321710

[12] Shigemoto N, Fukuda S, Takao S, Shimazu Y, Tanizawa Y, Kuwayama M, Ohara S. 2010. [Rapid detection of novel influenza A virus and seasonal influenza A (H1N1, H3N2) viruses by reverse transcription-loop-mediated isothermal amplification (RT-LAMP)]. Kansenshogaku Zasshi 84:431–436. (In Japanese.) doi:10.11150/kansenshogakuzasshi.84.431. 20715552

[13] Wang D, Yu J, Wang Y, Zhang M, Li P, Liu M, Liu Y. 2020. Development of a real-time loop-mediated isothermal amplification (LAMP) assay and visual LAMP assay for detection of African swine fever virus (ASFV). J Virol Methods 276:113775. doi:10.1016/j.jviromet.2019.113775. 31726114

[14] Wu C, Zeng Y, He Y. 2021. Rapid visualization and detection of Staphylococcus aureus based on loop-mediated isothermal amplification. World J Microbiol Biotechnol 37:209. doi:10.1007/s11274-021-03178-0. 34719733

[15] Ou H, Wang Y, Gao J, Bai J, Zhang Q, Shi L, Wang X, Wang C. 2021. Rapid detection of Salmonella based on loop-mediated isothermal amplification. Ann Palliat Med 10:6850–6858. doi:10.21037/apm-21-1387. 34237982

[16] Liu CY, Song HQ, Zhang RL, Chen MX, Xu MJ, Ai L, Chen XG, Zhan XM, Liang SH, Yuan ZG, Lin RQ, Zhu XQ. 2011. Specific detection of Angiostrongylus cantonensis in the snail Achatina fulica using a loop-mediated isothermal amplification (LAMP) assay. Mol Cell Probes 25:164–167. doi:10.1016/j.mcp.2011.04.002. 21515360

[17] Kong QM, Lu SH, Tong QB, Lou D, Chen R, Zheng B, Kumagai T, Wen LY, Ohta N, Zhou XN. 2012. Loop-mediated isothermal amplification (LAMP): early detection of Toxoplasma gondii infection in mice. Parasit Vectors 5:2. doi:10.1186/1756-3305-5-2. 22214421 PMC3280158

[18] Nakayama T, Yamazaki T, Yo A, Tone K, Mahdi Alshahni M, Fujisaki R, Makimura K. 2017. Detection of fungi from an indoor environment using loop-mediated isothermal amplification (LAMP) method. Biocontrol Sci 22:97–104. doi:10.4265/bio.22.97. 28659561

[19] Mauldin MR, McCollum AM, Nakazawa YJ, Mandra A, Whitehouse ER, Davidson W, Zhao H, Gao J, Li Y, Doty J, Yinka-Ogunleye A, Akinpelu A, Aruna O, Naidoo D, Lewandowski K, Afrough B, Graham V, Aarons E, Hewson R, Vipond R, Dunning J, Chand M, Brown C, Cohen-Gihon I, Erez N, Shifman O, Israeli O, Sharon M, Schwartz E, Beth-Din A, Zvi A, Mak TM, Ng YK, Cui L, Lin RTP, Olson VA, Brooks T, Paran N, Ihekweazu C, Reynolds MG. 2022. Exportation of monkeypox virus from the African continent. J Infect Dis 225:1367–1376. doi:10.1093/infdis/jiaa559. 32880628 PMC9016419

[20] Li Y, Olson VA, Laue T, Laker MT, Damon IK. 2006. Detection of monkeypox virus with real-time PCR assays. J Clin Virol 36:194–203. doi:10.1016/j.jcv.2006.03.012. 16731033 PMC9628957

[21] Li Y, Zhao H, Wilkins K, Hughes C, Damon IK. 2010. Real-time PCR assays for the specific detection of monkeypox virus West African and Congo Basin strain DNA. J Virol Methods 169:223–227. doi:10.1016/j.jviromet.2010.07.012. 20643162 PMC9628942

[22] Maksyutov RA, Gavrilova EV, Shchelkunov SN. 2016. Species-specific differentiation of variola, monkeypox, and varicella-zoster viruses by multiplex real-time PCR assay. J Virol Methods 236:215–220. doi:10.1016/j.jviromet.2016.07.024. 27477914 PMC9629046

[23] Quarleri J, Delpino MV, Galvan V. 2022. Monkeypox: considerations for the understanding and containment of the current outbreak in non-endemic countries. Geroscience doi:10.1007/s11357-022-00611-6. PMC9208705 35726117

[24] Iizuka I, Saijo M, Shiota T, Ami Y, Suzaki Y, Nagata N, Hasegawa H, Sakai K, Fukushi S, Mizutani T, Ogata M, Nakauchi M, Kurane I, Mizuguchi M, Morikawa S. 2009. Loop-mediated isothermal amplification-based diagnostic assay for monkeypox virus infections. J Med Virol 81:1102–1108. doi:10.1002/jmv.21494. 19382264

[25] Yuan J, Liu W, Huang L. 2014. A method used to prevent nucleic acid contamination and to indicate the result of nucleic acid thermostatic amplification reaction. China patent CN 201210371448.5.

