# Supplementary material for: Development of a Loop-Mediated Isothermal Amplification Method for Rapid and Visual Detection of Monkeypox Virus
Source: Microbiol Spectr. 2022 Sep 26;10(5):e02714-22. doi: 10.1128/spectrum.02714-22 (PMC9603857; doi:10.1128/spectrum.02714-22)
Supplement: Supplemental file 1 — Supplemental material. Download spectrum.02714-22-s0001.pdf, PDF file, 0.8 MB [file spectrum.02714-22-s0001.pdf]

Supplemental materials

Supplementary Table 1 (Table S1). Primer sets selected for loop-mediated isothermal amplification (LAMP) detection of monkeypox virus

Primer name	Primer sequence (5’-3’)
A27L-1F3	TTCTTGTATTTGTGGGAACAT
A27L-1B3	GATGGATGAGGAAGTGCC
A27L-1FIP	CCATCCCCCACCTAATAATGATAAATAGGATCTTCTAATGGATTGTATGG
A27L-1BIP	AATTGGTTGGTCCTCCTTATCTCCACAAGCATTTGTCTAAGCCTA
A27L-1LB	TCCAGTAGCATGTGGTTC
A27L-2F3	ACCTTAATTGGTTGGTCCTC
A27L-2B3	AGATACAGAAACAGAGTTCTTCT
A27L-2FIP	CGACAAGCATTTGTCTAAGCCTATTCTTATCTCCTCCAGTAGCATG
A27L-2BIP	GGCACTTCCTCATCCATCAAGTACTAACTCCTGAAGTCGCG
A27L-2LF	ACTAATACTGGTATTGAAGAACC
A27L-3F3	GGGGGATGGACAACCTTA
A27L-3B3	AGATACAGAAACAGAGTTCTTCT
A27L-3FIP	CGACAAGCATTTGTCTAAGCCTATTGTTGGTCCTCCTTATCTCC
A27L-3BIP	GGCACTTCCTCATCCATCAAGTACTAACTCCTGAAGTCGCG
A27L-3LF	GTATTGAAGAACCACATGCT
A27L-4F3	CTAATGGATTGTATGGCTTGA
A27L-4B3	TCTGAAACAGAACCCGATT

A27L-4FIP	GGAGATAAGGAGGACCAACCAATTAGCATCATCTTTATCATTATTAGG TG
A27L-4BIP	TCCAGTAGCATGTGGTTCTTCAATAGATGGATGAGGAAGTGCC
A27L-4LF	AGGTTGTCCATCCCC
A27L-4LB	ATTAGTAATAGGCTTAGACAAATGC
A27L-5F3	TGGCTTGATAGCATCATCT
A27L-5B3	CGTCTCTTAGAAAAGAATATTCTGA
A27L-5FIP	CTACTGGAGGAGATAAGGAGGACTATCATTATTAGGTGGGGGATG
A27L-5BIP	AATAGGCTTAGACAAATGCTTGTCGCCCGATTATAAATACTTGATGGAT G
A27L-5LB	TACGCGGGCACTTCC
F3L-1F3	TCTCGTTTAGATTTTCCATCTG
F3L-1B3	TCTTTTGATGATGTTATTCCGG
F3L-1FIP	TGGGGCCTAGTAACTCTCCTACCCTTATCGAATACTCTTCCGT
F3L-1BIP	TCAATACGAAAAGACCAATCTCTCCAAAGGTGTTAACCCTGTCAC
F3L-1LF	ATTTTATGCCTGTGTAGACATTG
F3L-2F3	TTTCCATCTGCCTTATCGA
F3L-2B3	TCTTTTGATGATGTTATTCCGG
F3L-2FIP	TGAATCAGTGGGGCCTAGTAACCTCTTCCGTCAATGTCTACAC
F3L-2BIP	TACGAAAAGACCAATCTCTCCTAGTTTGGAAAGGTGTTAACCCTG
F3L-2LB	TATTTGGCAGTACTCATTAATAACGG
F3L-3F3	CAGCCAATTTAGCTGCATTA

F3L-3B3	TTAACCCTGTCACCGTTAT
F3L-3FIP	TGTGTAGACATTGACGGAAGAGTATTTTAGCATCTCGTTTAGATTTTCC
F3L-3BIP	AATGTAGGAGAGTTACTAGGCCCTGAGTACTGCCAAATAACTAGGA
F3L-3LB	CACTGATTCAATACGAAAAGAC
F3L-4F3	CTCTCCTAGTTATTTGGCAGTAC
F3L-4B3	ACGACAATGGATGCTGAT
F3L-4FIP	AGTCTTTTGATGATGTTATTCCGGTCATTAATAACGGTGACAGGGT
F3L-4BIP	TTATCCTCTCTCATTGATTTTTCGCACGGCCTACAGATTCTGAT
F3L-4LB	GGGATACATCATCTATTATAGCATC
F3L-5F3	AAAAGACCAATCTCTCCTAGT
F3L-5B3	ACGACAATGGATGCTGAT
F3L-5FIP	AGTCTTTTGATGATGTTATTCCGGTTGGCAGTACTCATTAATAACGGT
F3L-5BIP	TGATCCTCTCTCATTGATTTTTCGCACGGCCTACAGATTCTGAT
F3L-5LF	AAAGGTGTTAACCCTGTC

F3: outer forward primer; B3: outer backward primer; FIP: forward inner primer; BIP: backward inner primer; LF: loop forward primer; LB: loop backward primer; FIP: F1c-F2; BIP: B1c-B2

Supplementary figure 1

Five primer pairs for conventional PCR were selected and tested for their abilities to detect the *A27L* gene (A27L-1F3 and A27L-1B3, A27L-2F3 and A27L-2B3, A27L-3F3 and A27L-3B3, A27L-4F3 and A27L-4B3, A27L-5F3 and A27L-5B3) and the *F3L* gene (F3L-1F3 and F3L-1B3, F3L-2F3 and F3L-2B3, F3L-3F3 and F3L-3B3, F3L-4F3 and F3L-4B3, F3L-5F3 and F3L-5B3) of MPXV pseudovirus. As shown in Fig. S1, the primer pairs A27L-1F3 and A27L-1B3, F3L-1F3 and F3L-1B3 yield excellent amplification effect under the same reaction temperature (60℃), 54℃, 56℃, 58℃ and 60℃ showed same amplification performance, and 60℃ was chosen as the reaction temperature for subsequent experiment of conventional PCR.

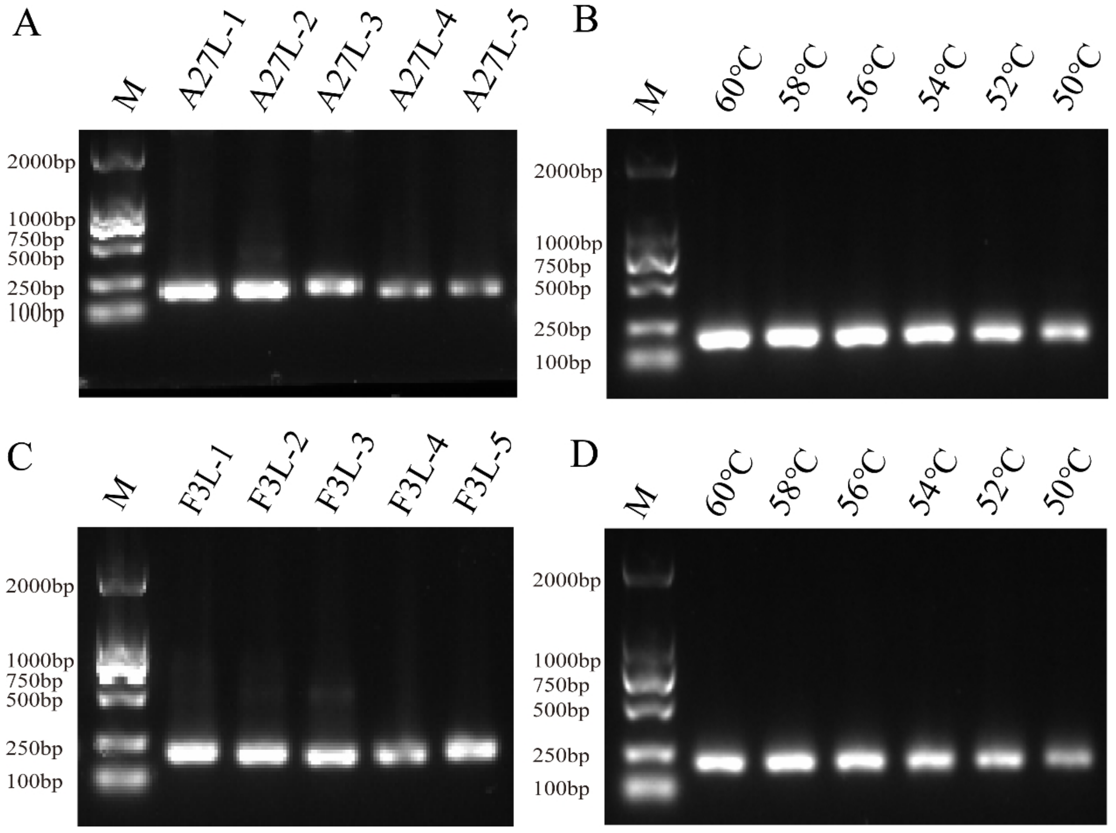

Fig. S1. Analysis of optimal primer and reaction temperature for conventional PCR assay of *A27L* and *F3L* genes of MPXV pseudovirus. (A) The best primer set for amplification of the *A27L* gene using conventional PCR assay. (B) The optimal reaction temperature for the conventional PCR assay with the A27L-1 primer set. (C) The best primer set for amplifying *F3L* using the conventional PCR assay. (D) The optimal reaction temperature for the conventional PCR assay with the primer set F3L-1. M: Marker.

Supplementary figure 2

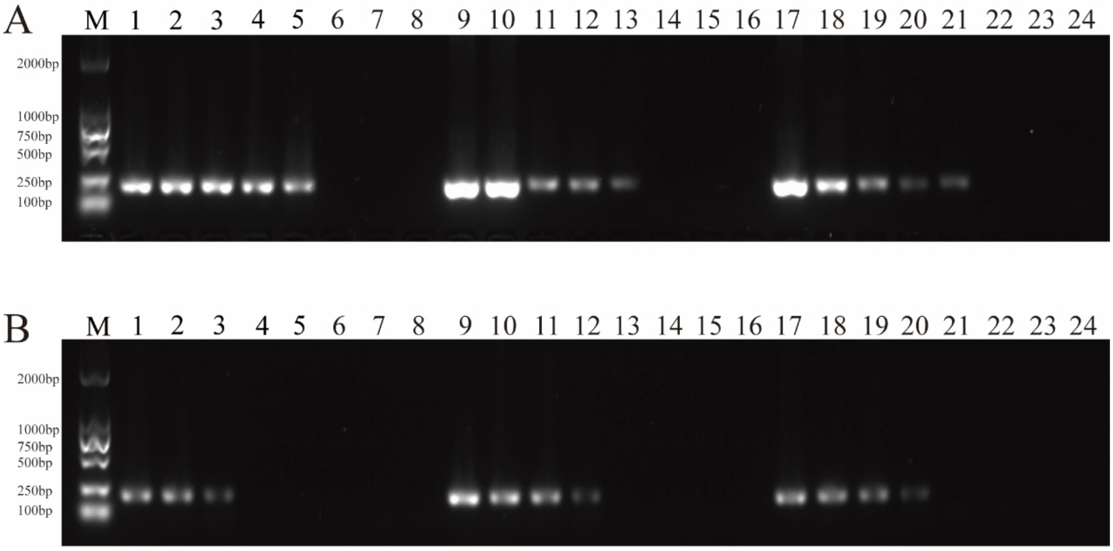

Fig. S2. Conventional PCR assay of simulated clinical samples. (A) The *A27L* gene detection of throat swab, peripheral blood and skin swab simulated clinical samples using conventional PCR assay. (B) The *F3L* gene detection of throat swab, peripheral blood and skin swab simulated clinical samples using conventional PCR assay. M: Marker; lines 1-8: throat swab simulated clinical samples 10^7, 10^6, 10^5, 10^4, 10^3, 10^2, 10^1, 10^0 copies/μL; lines 9-16: peripheral blood simulated clinical samples 10^7, 10^6, 10^5, 10^4, 10^3, 10^2, 10^1, 10^0 copies/μL; lines 17-24: skin swab simulated clinical samples 10^7, 10^6, 10^5, 10^4, 10^3, 10^2, 10^1, 10^0 copies/μL.

edanz 理文编辑
INNOVATIVE SCIENTIFIC SOLUTIONS

Certificate of Editing

Edited provisional title
Development of a loop-mediated isothermal amplification method for rapid and visual detection of monkeypox virus

Client name and institution
Junxia Feng , Capital Institute of Pediatrics, NO2, Yabao Road, Chaoyang District, Beijing, 100020, China.

Date Completed
2022-08-16

Identification code
110125

Certificate issued by
Koji Yamashita
Managing Director and CEO

Expert Editor: Katherine Thieltges
2008 Master of Public Health
Oregon State University
Medicinal and Biomolecular Chemistry, Biochemistry and Cell Biology, Public Health and Health Services

www.liwenbianji.cn
While this certificate confirms the authors have used Edanz's editing services, we cannot guarantee that additional changes have not been made after our edits.

edanz 理文编辑
INNOVATIVE SCIENTIFIC SOLUTIONS

Certificate of Editing

Edited provisional title

Development of a loop-mediated isothermal amplification method for rapid and visual detection of monkeypox virus

Client name and institution

Junxia Feng, Capital Institute of Pediatrics, NO2, Yabao Road, Chaoyang District , Beijing , 100020, China.

Date Completed
2022-06-30

Identification code
108798

Certificate issued by
Koji Yamashita
Managing Director and CEO

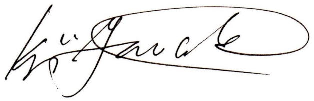

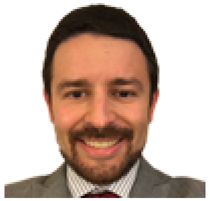

Expert Editor: Ryan Chastain-Gross
2015 PhD Medical Sciences
University of Florida
Dentistry, Ophthalmology and Optometry, Immunology

www.liwenbianji.cn

While this certificate confirms the authors have used Edanz's editing services, we cannot guarantee that additional changes have not been made after our edits.
